# A three-parameter complete blood count triage tool for monocytosis to prioritize hematology evaluation: development in MIMIC-IV and external validation in two independent cohorts

**DOI:** 10.3389/fmed.2026.1902149

**Published:** 2026-09-25

**Authors:** Lingyun Xu, Jing Wu

**Affiliations:** 1 Department of Hematology, Fuyang People’s Hospital (The Affiliated Fuyang People’s Hospital of Anhui Medical University), Fuyang, Anhui, China; 2 Department of Hematology, The Second Affiliated Hospital of Chengdu Medical College, China National Nuclear Corporation 416 Hospital, Chengdu, China

**Keywords:** acute myeloid leukemia, complete blood count, external validation, hematology triage, monocytosis, myelodysplastic syndromes

## Abstract

**Introduction:**

Monocytosis is a common complete blood count (CBC) abnormality, but in non-hematology settings it may be difficult to distinguish reactive causes from patterns warranting urgent evaluation for acute myeloid leukemia (AML) or myelodysplastic syndromes (MDS). We aimed to develop and externally validate a simple, report-level CBC triage model to prioritize hematology assessment in patients with monocytosis.

**Methods:**

MonoxGB was developed using admission-level data from MIMIC-IV v2.2 and three routinely reported CBC variables: absolute monocyte count, monocyte percentage, and platelet count. No proprietary analyzer signals, scattergram-derived parameters, morphology flags, or manufacturer-specific cell-population data were used. External validation included a retrospective Fuyang cohort of 91 eligible patients with monocytosis, all of whom underwent bone marrow examination, and an eICU Collaborative Research Database cohort of 73,650 intensive care admissions with monocytosis.

**Results:**

In fivefold cross-validation within MIMIC-IV, the mean area under the receiver operating characteristic curve (AUC) was 0.931 ± 0.022. In the Fuyang cohort, 29 of 91 patients had AML/MDS; the AUC was 0.840 (95% confidence interval, 0.753–0.915), and average precision was 0.682. In eICU-CRD, the AUC was 0.886 (95% confidence interval, 0.839–0.928). At an exploratory local high-sensitivity threshold of 0.0028, sensitivity was 0.966, specificity was 0.500, and negative predictive value was 0.969. Across cohorts, AML/MDS was associated with a higher monocyte burden and lower platelet counts; platelet count monocyte percentage, and absolute monocyte count were the dominant model features.

**Discussion:**

MonoxGB is a three-parameter, CBC report-based triage tool that may help non-hematologists prioritize patients with monocytosis for earlier smear review or hematology assessment. It is not a diagnostic substitute for bone marrow evaluation or specialist judgment. Prospective workflow-based validation using a prespecified deployment threshold is required before routine implementation.

## 1 Introduction

Monocytosis is a common finding in routine complete blood count (CBC) tests. In most patients, it reflects reactive or transient conditions; however, it can also be an early clue to clonal myeloid disease, including acute myeloid leukemia (AML) and myelodysplastic syndromes (MDS) (1, 2). AML and MDS may initially present with a commonly described marrow-failure triad: fatigue or dyspnea related to anemia, infection or fever related to dysfunctional or reduced neutrophils, and bruising or bleeding associated with thrombocytopenia. However, this triad is neither universal nor diagnostic (3–5). CBC abnormalities and peripheral smear findings initiate the investigation, while definitive classification requires integrated morphologic, immunophenotypic, cytogenetic, and molecular assessments (4, 5). The early clinical problem is not merely the presence of monocytosis, but whether the initial CBC pattern is concerning enough to justify an earlier manual smear review, hematology consultation, or diagnostic escalation.

This question is especially important outside hematology practices and in CBC-equipped but hematology-expertise-limited settings. Monocytosis is often first identified in emergency departments, general inpatient wards, perioperative evaluations, and hospitals without on-site hematopathological support. Prior CBC-based screening studies have shown that combinations of routine indices, cytopenias, or research analyzer parameters can enrich for MDS or distinguish hematologic malignancies, with reported area under the receiver operating characteristic curves (AUCs) generally in the mid-to-high 0.80s and even higher when molecular or specialized analyzer variables are included (6–10). However, several published tools depend on research-use-only cell population data, analyzer-specific parameters, or cohorts selected for cytopenia, limiting their direct transfer to a simple report-level monocytosis workflow. Delayed recognition is important because treatment delay in AML may adversely affect outcomes, and acute-care pathways may be disrupted when an occult myeloid neoplasm is recognized late (11, 12).

Platelet count was included because the clinical signal of concern is not monocytosis in isolation, but monocytosis accompanied by evidence of disturbed marrow hematopoiesis. Thrombocytopenia can reflect ineffective hematopoiesis, marrow replacement, or treatment-independent marrow failure in AML and MDS, whereas many reactive causes of isolated monocytosis do not produce the same degree of platelet suppression (3–5). Platelet count is not specific for clonal disease and is not causally linked to monocytosis; rather, its coexistence with increased AMC or monocyte percentage provides complementary information. Consistent with prior CBC-based MDS screening studies, platelet-related information can help distinguish a broader clonal marrow pattern from isolated monocytosis (9, 10). Accordingly, we designed MonoxGB as a compact triage tool based only on AMC, monocyte percentage, and platelet count, rather than proprietary analyzer signals or molecular data. The aim was to develop the model in a large real-world cohort, externally validate it in two substantially different clinical environments, and test whether a deliberately high-sensitivity operating point could support prioritization for hematology assessment without presenting the model as a standalone diagnostic test.

## 2 Materials and methods

### 2.1 Study design, reporting, and ethics

This retrospective triage model study included one development cohort and two external validation cohorts. This study was reported with reference to the STARD 2015 and TRIPOD statements (13, 14). The local retrospective cohort study was approved by the Ethics Committee of Fuyang People's Hospital (The Affiliated Fuyang People's Hospital of Anhui Medical University; approval No. [2025]96; approved on April 23, 2025). Because the local analysis used de-identified retrospective clinical data, the requirement for written informed consent was waived by the ethics committee. Access to MIMIC-IV and eICU-CRD was obtained using their respective data-use frameworks.

### 2.2 Data sources and participant selection

The development cohort was derived from MIMIC-IV v2.2, a de-identified electronic health record database from Beth Israel Deaconess Medical Center (15). In MIMIC-IV, all eligible hospital admissions were included. For each hospitalization, only the earliest qualifying CBC was retained as an index measurement. We did not conduct separate analyses of first admissions and all admissions. Eligible admissions were required to have monocytosis on the index CBC, defined as AMC >0.60 × 10^9^/L, together with available white blood cell count, monocyte percentage, and platelet count. Outcome labels for AML/MDS were derived from the database diagnosis sources, as specified in the Supplementary Appendix.

The first external validation cohort comprised 91 eligible patients with monocytosis admitted to Fuyang People's Hospital between 1 January 2021 and 31 December 2021. Potentially eligible patients were identified from the local clinical dataset using AMC >0.60 × 10^9^/L. The qualifying CBC was the first CBC obtained within 24 h of admission and before disease-directed therapy, blood transfusion, or exposure to glucocorticoids. Records with AMC ≤0.60 × 10^9^/L, missing required predictor measurements, acute promyelocytic leukemia (M3), or acute leukemia without confirmed myeloid lineage were excluded. All 91 patients underwent bone marrow examination, and the final clinician-adjudicated reference diagnosis comprised 29 AML/MDS and 62 non-AML/MDS patients.

The second external validation cohort was derived from the eICU Collaborative Research Database v2.0 (16). For eICU-CRD, the first qualifying CBC from each patient's first ICU admission was retained. The final eICU monocytosis cohort comprised 73,650 first ICU admissions: 92 AML/MDS-positive admissions (0.12%) and 73,558 non-AML/MDS admissions (99.88%). AML/MDS labels were defined from the diagnosis field using descriptions consistent with acute myeloid leukemia or myelodysplastic syndrome.

### 2.3 Intended clinical use, index test, and predictor variables

The intended use of MonoxGB was for clinical triage rather than as a standalone diagnostic tool. Specifically, the model was designed as a CBC report-based prioritization signal for patients in whom monocytosis had already been detected, to help identify which patients should undergo earlier manual smear review, hematology consultation, diagnostic escalation, or transfer. Predictor selection was clinically driven. Through long-term hematology consultation and the assessment of both outpatient and hospitalized patients, the investigators repeatedly observed that abnormalities in monocyte burden and platelet count were prominent in the CBC profiles of patients subsequently diagnosed with AML or MDS. AMC, monocyte percentage, and platelet count were therefore selected *a priori* based on clinical experience, routine availability, and biological plausibility. In the initial conceptual stage, a monocyte-to-platelet ratio was considered as a possible simplified index but was not pursued as a separate predictor or competing model. The final three-variable gradient boosting model retains the original values and can represent their joint and non-linear effects. Collapsing them into a fixed ratio would discard information and introduce a largely redundant derived variable. No broad stepwise, recursive, or data-driven screening of a larger set of candidate variables was performed; consequently, there are no formal “discarded-variable” performance results to report. In MIMIC-IV and eICU-CRD, AMC was calculated by multiplying the white blood cell count by the monocyte fraction, as required by the source schema. In the local cohort, predictor values were taken directly from the index CBC measured on the Mindray BC-6900 platform.

### 2.4 Report-level CBC variables, harmonization, and analyzer considerations

The three predictors were the standard report-level CBC values. No proprietary analyzer raw signals, scattergram-derived parameters, morphological flags, or manufacturer-specific cell population data were used as model inputs. AMC was considered a CBC-derived value, and when it was not directly available in the database schema, it was calculated by multiplying the total white blood cell count by the monocyte fraction using harmonized units. In the Fuyang cohort, CBC testing was performed on the Mindray BC-6900. According to the manufacturer, the WBC differential uses SF Cube three-dimensional cell analysis, integrating forward-scattered light, side-scattered light, and fluorescence signals to distinguish leukocyte populations. Red blood cell (RBC) and platelet enumeration use a secondary-accelerated sheath-flow direct-current impedance method. The analyzer can also obtain an optical platelet count (PLT-O) in the reticulocyte channel to cross-check the impedance platelet result and reduce interference from small red cells or large platelets (17). The present model used only the final report-level monocyte and platelet results, excluding the underlying optical signals or research parameters. The model requires a validated CBC with a reliable leukocyte differential and platelet count and should not be applied where only limited or poorly validated differential counts are available. Because the analyzer model, calibration, reagent system, software version, and flagging information were unavailable in the public databases, inter-analyzer variation could not be directly quantified. MonoxGB should therefore be interpreted as a report-level triage model that requires local analytical validation and threshold recalibration before deployment, rather than as a universally device-independent diagnostic assay.

### 2.5 Target condition and reference standards

The target condition was AML or MDS, analyzed jointly as AML/MDS in line with contemporary myeloid neoplasm classification frameworks (11). Clinically, monocytosis may also indicate potential concerns for chronic myelomonocytic leukemia, MDS/myeloproliferative neoplasm overlap neoplasms, AML with monocytic differentiation, and other clonal myeloid disorders. However, the reliable ascertainment of these additional entities was limited in the available public databases. Therefore, AML/MDS was used as a pragmatic endpoint for urgent clonal myeloid disease rather than as a complete representation of all clinically important causes of monocytosis. In the expanded Fuyang cohort, the reference standard was the clinician-adjudicated final diagnosis established by bone marrow examination. In MIMIC-IV and eICU-CRD, reference labels were derived from coded or curated diagnostic data.

### 2.6 Model development and feature-contribution analysis

The final model was a gradient boosting classifier implemented in Scikit-learn (18, 19). Gradient boosting was selected because it can capture non-linear relationships and variable interactions while remaining computationally lightweight enough for potential deployment in laboratory or electronic medical record workflows. Model development used only the three prespecified report-level CBC predictors. SHapley Additive exPlanations (SHAP) summarizes feature contributions and directional effects (20). Because SHAP values are model explanations rather than independently sampled biological outcomes, no conventional null hypothesis test was used to assign a *P*-value to the feature rank. Instead, the uncertainty in the mean absolute SHAP values was quantified using 2,000 bootstrap resamples of the reproducible 5,000-admission explanation sample, with 95% confidence intervals. The model itself is referred to hereafter as MonoxGB, but the clinical emphasis is on its function as a CBC-based triage tool rather than its algorithmic architecture.

### 2.7 Statistical analysis

Internal validation in the development cohort used fivefold stratified cross-validation and was reported as the mean AUC ± standard deviation across folds. Pooled out-of-fold predictions were also used to construct the development receiver operating characteristic (ROC) and precision-recall curves and for formal cross-cohort AUC comparison. Apparent development cohort performance was retained for descriptive context only. All ROC curves and AUCs were calculated in Python using the roc_curve and roc_auc_score functions in Scikit-learn (19). In the two external cohorts, we assessed the AUC, precision-recall performance, Brier score, and descriptive threshold-dependent metrics. For each cohort, the statistically optimal cutoff was defined as the predicted probability maximizing Youden J (sensitivity + specificity − 1) on that cohort. Pairwise AUC differences among the three independent cohorts were tested using two-sided *z-*tests based on DeLong variance estimates, with 95% confidence intervals and Holm adjustment across the three comparisons (21). In the expanded Fuyang cohort, an additional exploratory high-sensitivity operating point of 0.0028 was selected *post hoc* to achieve a sensitivity of ≥0.95 for the intended screening use. This high-sensitivity operating point was reported separately from the Fuyang Youden-optimal cutoff. Decision curve analysis was used to explore the potential clinical utility (22). As shown in Figure 1, pooled outcome group distributions were compared exploratorily using two-sided Mann–Whitney *U* tests, with absolute rank-biserial correlation as the effect size. These unadjusted pooled comparisons were interpreted descriptively because cohort heterogeneity can influence the distributions. No formal sample size calculation was performed, and cohort sizes were determined based on the available eligible records.

**Figure 1:**
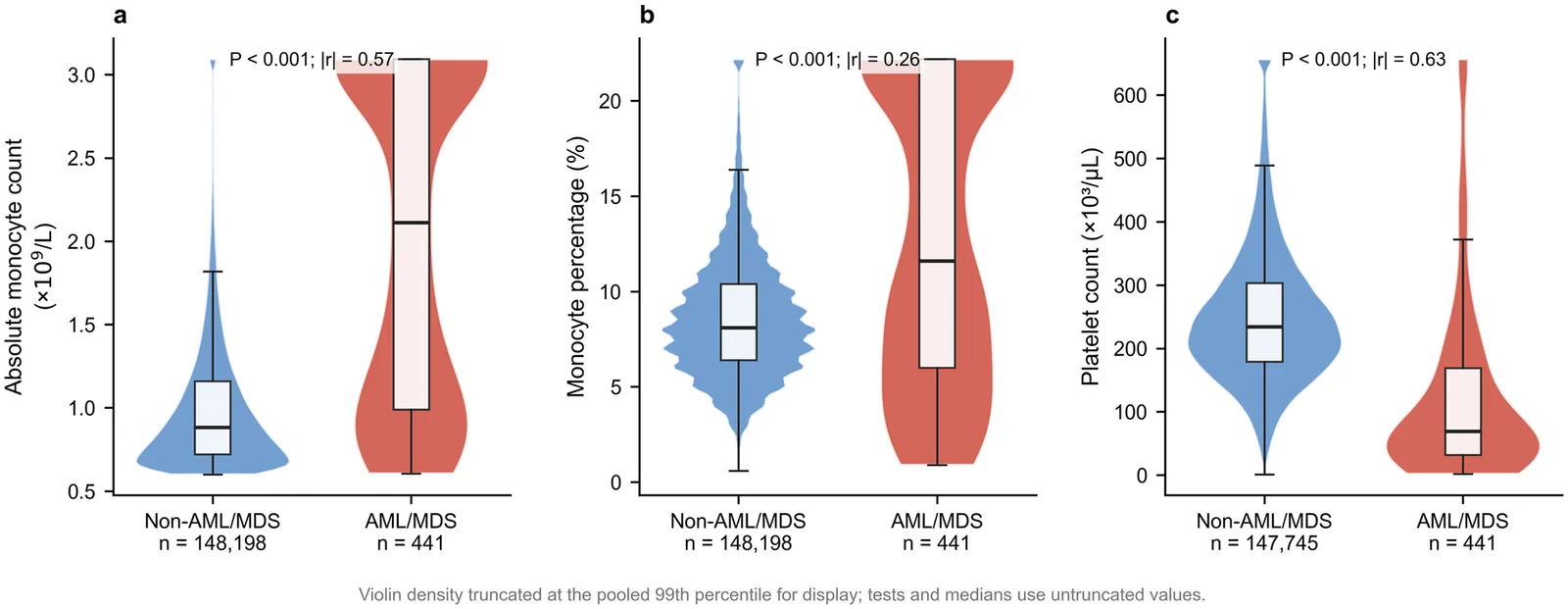
Outcome-group distributions of **(a)** absolute monocyte count (AMC), **(b)** monocyte percentage, and **(c)** platelet count pooled across cohorts. Violin shapes display distributions truncated at the feature-specific 99th percentile for visualization; the embedded boxes show the median and interquartile range from the untruncated data. Two-sided Mann–Whitney U *P*-values and absolute rank-biserial correlations are exploratory, unadjusted pooled comparisons. Sample sizes are printed in each panel, and platelet data were available for 147, 745 non-AML/MDS observations and 441 AML/MDS observations.

## 3 Results

### 3.1 Cohort characteristics

The study flow is shown in Figure 2. The development cohort included 74,898 MIMIC-IV admissions with monocytosis, of which 320 (0.43%) were AML/MDS-positive. The expanded local external cohort included 91 patients, of whom 29 (31.87%) had AML/MDS and 62 had non-AML/MDS. The eICU external cohort included 73,650 ICU admissions with monocytosis, of which 92 (0.12%) were AML/MDS-positive.

**Figure 2:**
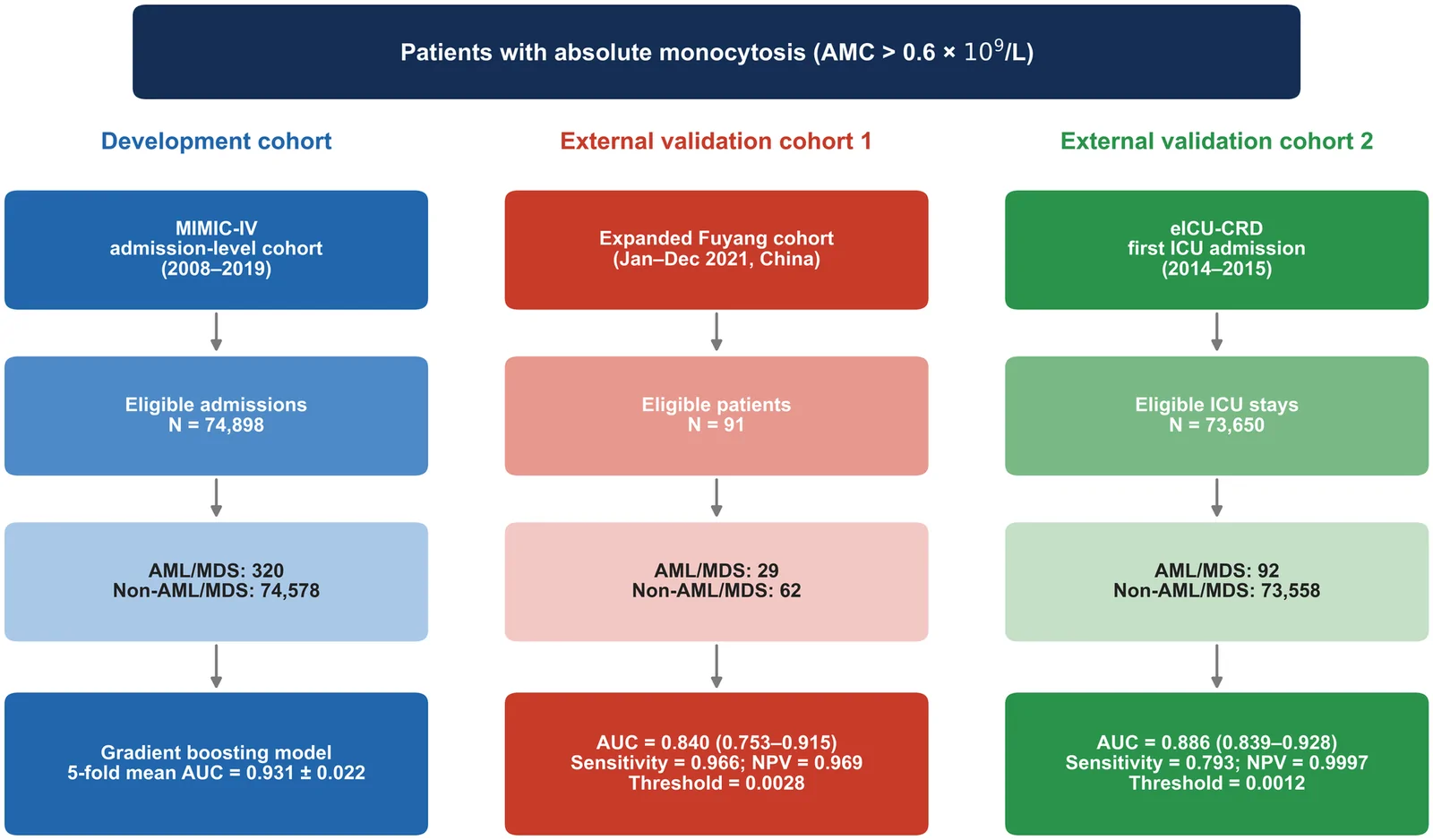
Study design and participant flow diagram. The development used admission-level MIMIC-IV data, with the earliest qualifying CBC within each eligible hospitalization. External validation used the expanded Fuyang People's Hospital cohort (1 January to 31 December 2021; *N* = 91; all underwent bone marrow examination) and the first ICU admission per patient in eICU-CRD. External cohort sensitivity and negative predictive values are shown at the descriptive thresholds specified in the Methods section.

Across all three cohorts, AML/MDS-positive patients had a pattern of higher monocyte burden and lower platelet count compared to comparator patients (Table 1). In the expanded Fuyang cohort, the median AMC was 3.26 × 10^9^/L in AML/MDS versus 0.97 × 10^9^/L in non-AML/MDS, the median monocyte percentage was 32.5% versus 9.3%, and the median platelet count was 38.0 × 10^3^/μL versus 135.5 × 10^3^/μL. A similar directional separation was observed in the MIMIC-IV and eICU-CRD, although the absolute distributions differed across the settings.

**Table 1:** Baseline complete blood count characteristics by cohort and outcome group.

Cohort	*N*	AML/MDS *n* (%)	AMC, non-AML/MDS	AMC, AML/MDS	Monocyte %, non-AML/MDS	Monocyte %, AML/MDS	Platelets, non-AML/MDS	Platelets, AML/MDS
MIMIC-IV development cohort	74,898	320 (0.43%)	0.85 [0.71–1.10]	2.10 [1.01–5.27]	8.3 [6.4–10.7]	10.9 [5.0–23.0]	237.0 [178.0–311.0]	70.5 [35.0–165.0]
Expanded Fuyang cohort	91	29 (31.87%)	0.97 [0.74–1.46]	3.26 [1.45–23.37]	9.3 [4.2–13.2]	32.5 [20.9–58.3]	135.5 [41.5–343.0]	38.0 [22.0–80.0]
eICU-CRD external cohort	73,650	92 (0.12%)	0.92 [0.74–1.22]	1.74 [0.90–5.88]	8.0 [6.3–10.0]	11.0 [6.8–23.0]	231.0 [179.0–296.0]	72.0 [29.8–215.0]

Values are median [interquartile range]. The comparator group denotes non-AML/MDS admissions or patients. In the corrected expanded Fuyang cohort, all 91 included patients underwent bone marrow examination, and groups were based on the clinician-adjudicated final diagnosis. AMC indicates absolute monocyte count (×10^9^/L); platelet counts are reported as ×10^3^/μL.

### 3.2 Discrimination performance

In the fivefold cross-validation within MIMIC-IV, MonoxGB achieved a mean AUC of 0.931 ± 0.022. The pooled out-of-fold AUC was 0.923, with an average precision (AP) of 0.199; these predictions, rather than apparent fitted probabilities, were used for the development curves and cross-cohort AUC comparisons. The apparent development cohort AUC was 0.945, with an average precision of 0.461, and was reported only as a descriptive context (Table 2; Figure 3).

**Table 2:** Performance summary of MonoxGB in the development and external validation datasets.

Cohort	AUC (95% CI)	AUPRC/AP	Brier score	Threshold	Sensitivity	Specificity	PPV	NPV
MIMIC-IV pooled out-of-fold	0.923	0.199	NR	0.00218 (Youden)	0.859	0.920	0.044	0.9993
MIMIC-IV 5-fold cross-validation	0.931 ± 0.022	NR	NR	Not prespecified	—	—	—	—
MIMIC-IV apparent (descriptive only)	0.945	0.461	0.003	Descriptive only	—	—	—	—
Expanded Fuyang cohort, high-sensitivity	0.840 (0.753–0.915)	0.682	0.206	0.0028	0.966	0.500	0.475	0.969
Expanded Fuyang cohort, Youden	0.840 (0.753–0.915)	0.682	0.206	0.02455	0.862	0.677	0.556	0.913
eICU-CRD	0.886 (0.839–0.928)	0.106	0.001	0. 001239	0.793	0.895	0.009	0.9997

AUPRC/AP, area under the precision–recall curve/average precision; PPV, positive predictive value; NPV, negative predictive value; NR, not reported. The Fuyang threshold of 0.0028 was selected *post hoc* as an exploratory high-sensitivity operating point and is reported separately from the cohort-specific Youden-optimal cut-off of 0.02455. The eICU-CRD threshold was cohort-specific and Youden-optimal. None of these thresholds is a locked deployment threshold. Pairwise DeLong comparisons were nonsignificant after Holm adjustment (all adjusted *P* ≥ 0.160), which does not establish equivalence.

**Figure 3:**
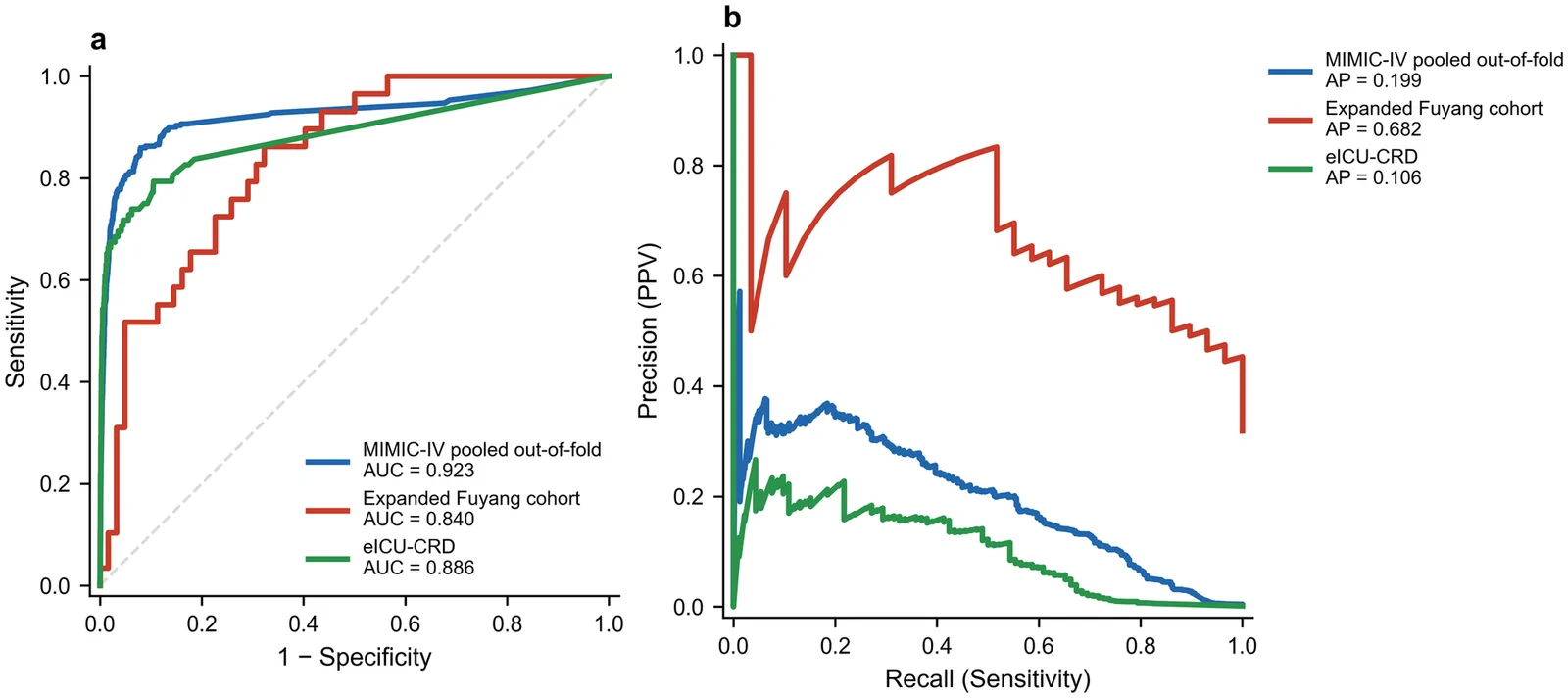
Discrimination performance in the development and external validation cohorts. **(a)** shows the receiver operating characteristic curves, and **(b)** shows the precision-recall curves. The MIMIC-IV curves use pooled out-of-fold predictions (AUC: 0.923; AP: 0.199); external curves show the expanded Fuyang cohort (AUC: 0.840; AP: 0.682) and eICU-CRD (AUC: 0.886; AP: 0.106). The legends are repositioned to avoid obscuring the curves.

External discrimination remained strong in both validation cohorts. In the expanded Fuyang cohort, the AUC was 0.840 (95% CI: 0.753–0.915) and the average precision was 0.682. In eICU-CRD, the AUC was 0.886 (95% CI: 0.839–0.928), and the average precision was 0.106. No pairwise AUC difference remained statistically significant after Holm adjustment: MIMIC-IV pooled out-of-fold versus Fuyang, difference 0.083 (95% CI: −0.001 to 0.168; adjusted *P* = 0.160); MIMIC-IV versus eICU-CRD, difference 0.037 (95% CI: −0.012 to 0.087; adjusted *P* = 0.273); and Fuyang versus eICU-CRD, difference −0.046 (95% CI: −0.139 to 0.047; adjusted *P* = 0.332). These nonsignificant tests do not establish equivalence. The lower precision-recall performance in the eICU reflects the very low prevalence of AML/MDS rather than a collapse in ranking performance.

### 3.3 Threshold-dependent performance

The Youden-optimal predicted probability cutoffs differed across cohorts. For MIMIC-IV pooled out-of-fold predictions, the optimal cutoff was 0.00218, yielding a sensitivity of 0.859 and a specificity of 0.920. For the expanded Fuyang cohort, the Youden-optimal cutoff was 0.02455, yielding a sensitivity of 0.862 and a specificity of 0.677. At the exploratory high-sensitivity threshold of 0.0028, sensitivity was 0.966, specificity was 0.500, positive predictive value was 0.475, and negative predictive value was 0.969. The confusion matrix comprised 28 true positives, 1 false negative, 31 true negatives, and 31 false positives. The single false-negative case was MDS. As a counterfactual workload scenario, rather than a measured comparison with historical referral practice, the tool would prioritize 59/91 patients (64.8%) for hematology assessment, capture 28/29 AML/MDS cases, and avoid immediate referral in 32/91 (35.2%) compared with a refer-all strategy. Neither threshold should be regarded as a locked deployment threshold.

### 3.4 Calibration, decision curve analysis, and feature effects

The Brier scores were 0.206 in the expanded Fuyang cohort and 0.001 in eICU-CRD. The calibration curves are shown in Figures 4a,b and should be interpreted in light of the modest local sample size and pronounced class imbalance in the eICU. The decision curve analyses are shown in Figures 4c,d. They suggested an apparent net benefit in the expanded Fuyang cohort across part of the displayed threshold range, whereas the eICU curve closely followed the treat-none strategy over much of the range, consistent with the rarity of the target condition in that setting.

**Figure 4:**
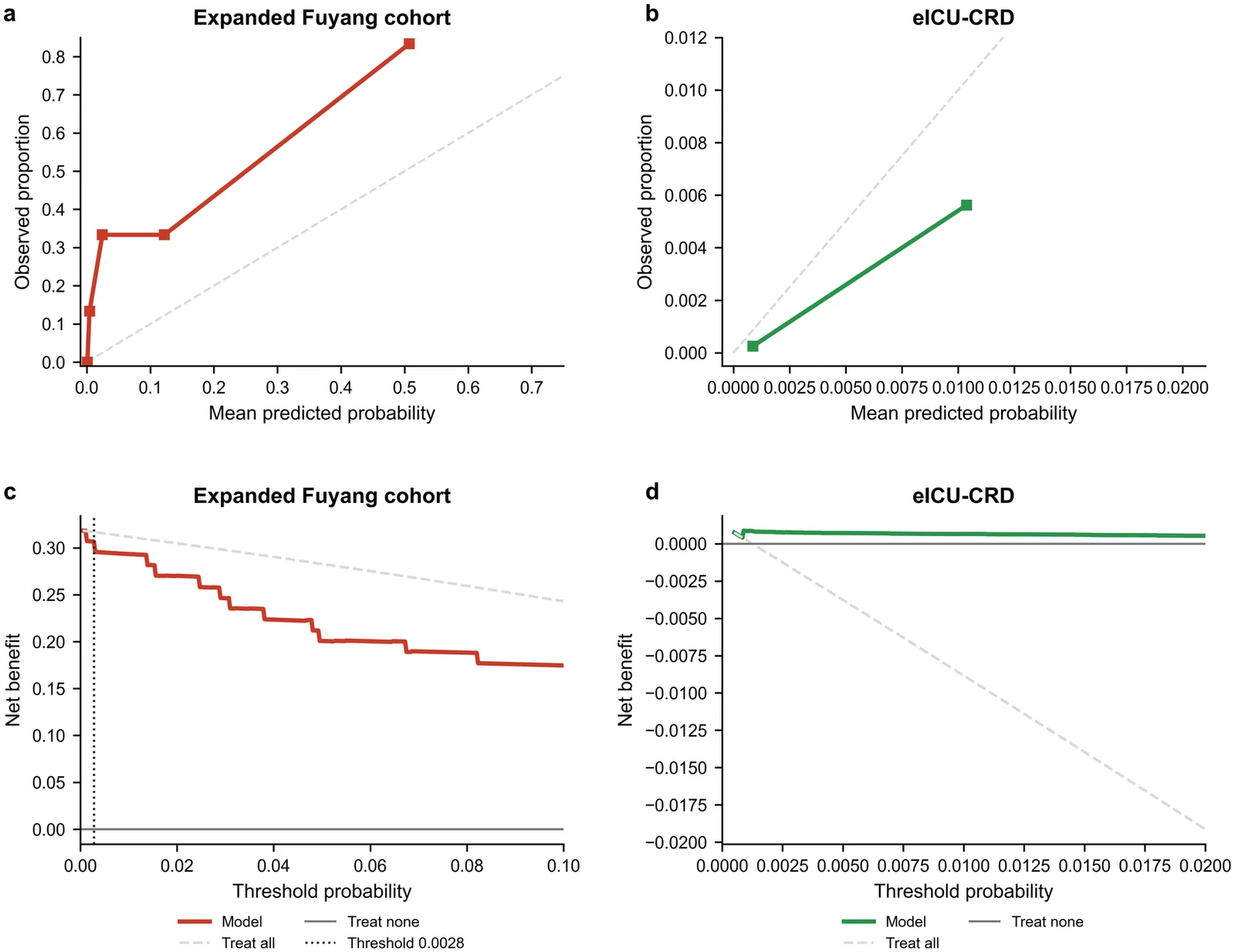
Calibration and decision curve analysis in the external validation cohort. **(a,b)** show the calibration in the expanded Fuyang cohort and eICU-CRD, respectively. **(c,d)** show the corresponding decision curve analyses. Legends are placed outside the principal curve region to avoid overlap.

Feature-contribution analysis ranked platelet count as the most influential predictor (mean absolute SHAP 0.227, bootstrap 95% CI: 0.213–0.242), followed by monocyte percentage (0.125, 95% CI: 0.117–0.134) and AMC (0.108, 95% CI: 0.099–0.118) (Figure 5). Lower platelet counts and higher monocyte burden measures shifted the predictions toward AML/MDS. Exploratory pooled comparisons in Figure 1 showed median AMC 2.112 versus 0.883 × 10^9^/L (*P* = 6.93 × 10^−94^; absolute rank-biserial *r* = 0.566), monocyte percentage 11.6% versus 8.1% (*P* = 4.23 × 10^−21^; *r* = 0.259), and platelet count 69 versus 234 × 10^3^/μL (*P* = 2.30 × 10^−115^; *r* = 0.629) in AML/MDS versus non-AML/MDS.

**Figure 5:**
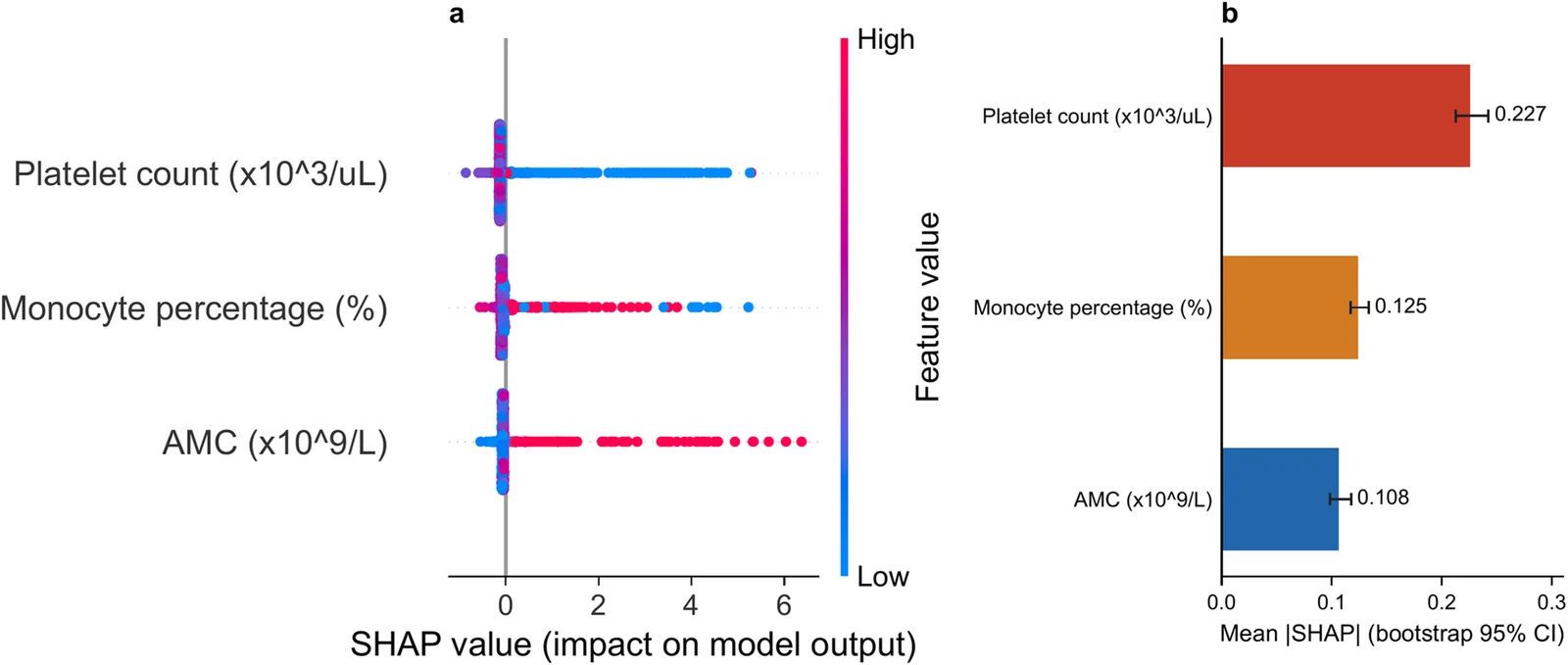
SHAP feature-contribution analysis. **(a)** shows the SHAP beeswarm plot for a reproducible 5,000-admission MIMIC-IV explanation sample. **(b)** shows the mean absolute SHAP values with 95% confidence intervals from 2,000 bootstrap resamples. The intervals quantify the uncertainty in the mean contribution magnitude; they are not null hypothesis significance tests of biological association.

## 4 Discussion

This study supports a clinically oriented view of MonoxGB. Its value lies not in the application of gradient boosting *per se*, but in translating three routinely reported CBC measurements into a triage signal for monocytosis. Across a large development cohort and two external validation cohorts, the model consistently identified a pattern of higher monocyte burden and thrombocytopenia associated with AML/MDS. This pattern is compatible with the biology and clinical presentation of myeloid neoplasms, in which ineffective or displaced hematopoiesis can produce cytopenia, while abnormal myeloid differentiation alters leukocyte composition (3–5).

The intended use of such a tool is upstream of the definitive diagnosis. Current AML and MDS frameworks require integrated morphologic, immunophenotypic, cytogenetic, and molecular evaluations; no three-variable CBC model can replace this pathway (4, 5). The relevant question is which patients with monocytosis should be prioritized for an earlier review. In practice, monocytosis is often first noted by non-hematologists who must decide whether repeat testing is sufficient or whether the overall CBC pattern warrants an accelerated smear review, hematology consultation, or transfer. This distinction is especially relevant when CBC testing is available, but timely hematopathology and marrow-based diagnostics are not.

MonoxGB complements, rather than duplicates, prior CBC-based screening tools. Boutault et al. combined the mean corpuscular volume, absolute neutrophil count, and an analyzer-derived neutrophil dispersion parameter to screen cytopenic patients for MDS, reporting a sensitivity of 86% and a specificity of 88% (9). Pozdnyakova et al. reported an AUC of 0.86 for a routine-CBC-only MDS model, which increased to 0.93 after adding molecular and demographic variables(6). Urrechaga et al. prospectively validated a model using research-use-only cell population data with an AUC of approximately 0.88 (7), while Haider et al. and Zhu et al. showed that research CBC parameters and machine learning can support leukemia differentiation or MDS smear review decisions (8, 10). MonoxGB addresses a more specifically targeted and clinically relevant population—patients already identified with monocytosis—and was deliberately kept simpler to facilitate triage, using only report-level AMC, monocyte percentage, and platelet count. Existing tools have relied on research-use-only cell population data, analyzer-specific parameters, or cohorts selected for cytopenia, and no previous study has specifically targeted patients with monocytosis. These features limit the direct translation of the published tools to a simple, report-level monocytosis workflow. The strongest SHAP contribution from platelet count, along with supportive contributions from both monocyte measures, also argues against interpreting MonoxGB as an AMC-only rule.

This study has several important limitations. First, although local enrollment covered the complete 2021 calendar year, the Fuyang cohort included only 91 patients (29 with AML/MDS and 62 with non-AML/MDS). The sample size remains limited, resulting in substantial uncertainty regarding the performance estimates and insufficient power for reliable disease subtypes or other small subgroup analyses. The principal reason for this limited sample size was that the selected cohort consisted of patients who had undergone bone marrow examination, which was required to establish a definitive diagnosis and evaluate the model's performance against an appropriate reference standard. Second, the local high-sensitivity threshold was selected *post hoc*, and each cohort-specific Youden cutoff was estimated within the same cohort in which it was reported; no threshold was independently locked and validated. Therefore, local deployment should use thresholds derived from preliminary local data. Third, the model targets urgent conditions represented by AML/MDS rather than a broader spectrum of clonal myeloid diseases. Fourth, the extremely low prevalence of eICU constrains the positive predictive value.

These limitations define future research directions. A prospective silent-deployment study should set a threshold before enrollment and record whether a hematology assessment, manual smear review, marrow examination, or transfer occurred under usual care and at what time for every eligible patient. The prespecified comparison should include the proportion referred by usual care versus flagged by MonoxGB, paired discordance testing where appropriate, sensitivity for marrow-confirmed AML/MDS, time to specialist review, false-positive workload, missed disease, and decision curve net benefit. Local analytical validation and threshold recalibration are necessary before implementation in a new laboratory environment.

In summary, MonoxGB should be considered a CBC-based triage tool for patients with monocytosis, intended to identify those who urgently require hematology management or consultation. It does not replace marrow evaluation, smear interpretation, flow cytometry, cytogenetic testing, molecular testing, or hematologist judgment. Instead, it offers a potentially scalable way to identify which patients with monocytosis may not be safely managed by watchful waiting alone and may merit earlier specialist assessment, particularly in CBC-equipped settings where hematology expertise is limited.

## Data Availability

The original contributions presented in this study are included in the article/Supplementary Material. Further inquiries can be directed to the corresponding author.
